# Clinicopathologic characteristics of early‐onset breast cancer among unselected young Black women

**DOI:** 10.1002/cncr.70402

**Published:** 2026-06-08

**Authors:** Heather K. Beasley, Tiana Shah, Rory J. Tinker, Anne Weidner, Lindsay Venton, Chunling Hu, Mya L. Roberson, Brian D. Lehmann, Fergus J. Couch, Sonya Reid, Kelly Metcalfe, Tuya Pal

**Affiliations:** ^1^ Department of Medicine Vanderbilt University Medical Center Nashville Tennessee USA; ^2^ Department of Genetics and Genomic Sciences Icahn School of Medicine at Mount Sinai New York New York USA; ^3^ Department of Laboratory Medicine and Pathology Mayo Clinic Rochester Minnesota USA; ^4^ Lineberger Comprehensive Cancer Center University of North Carolina at Chapel Hill Chapel Hill North Carolina USA; ^5^ Lawrence S. Bloomberg Faculty of Nursing University of Toronto Toronto Ontario Canada

**Keywords:** Black women, breast cancer, breast cancer age distribution, breast cancer characteristics, germline pathogenic/likely pathogenic variants, hereditary breast cancer, registry‐based study

## Abstract

**Background:**

Black women are more likely to develop and die from early‐onset breast cancer. Hereditary breast cancer (HBC) is strongly associated with early age of onset. Accordingly, HBC characteristics in young Black women were evaluated.

**Methods:**

Black women diagnosed with invasive breast cancer at age 50 years or younger between 2005 and 2018 were recruited through the state cancer registries in Florida and Tennessee. Participants were asked to complete a questionnaire, sign medical records releases, and donate a saliva sample for free genetic testing to identify HBC germline pathogenic or likely pathogenic variant (GPV). Women with sporadic versus hereditary breast cancer were compared, based on age distribution, clinical characteristics, and family cancer history.

**Results:**

Among 686 participants with confirmed genetic testing, 15.3% had GPVs in breast and/or ovarian cancer predisposition genes, including *BRCA1* (7.7%), *BRCA2* (4.5%), *PALB2* (0.9%), and *ATM* (0.7%), with 1.5% in other genes. Compared to the sporadic group, triple‐negative breast cancers were overrepresented among *BRCA1* carriers (23.6% vs. 75.5%; *p* < .001), and family history of breast cancer was significantly more frequent among *BRCA1*, *BRCA2*, and *PALB2* carriers (all *p* < .05). The majority of breast cancers occurred by age 40 years in *BRCA1* carriers (52.8%), compared to other genes, where age was more evenly distributed.

**Conclusion:**

Among unselected young Black women with breast cancer, our findings demonstrate that the highest frequency of HBC GPVs are in *BRCA1*, *BRCA2,* and *PALB2,* with identification of distinct histopathologic and demographic characteristics across HBC genes, underscoring the critical need for genetic testing in all populations.

## Abstract 2

In this study of 686 young Black women diagnosed with invasive breast cancer, 15.3% carried germline pathogenic or likely pathogenic variants in breast and/or ovarian cancer predisposition genes, with the highest frequencies in *BRCA1* (7.7%), *BRCA2* (4.5%), and *PALB2* (0.9%). Distinct histopathologic and demographic characteristics were identified across hereditary breast cancer genes, underscoring the critical need for genetic testing in all populations.

## INTRODUCTION

Black women experience disproportionately higher incidence and mortality from early‐onset breast cancer, in part because of higher prevalence of aggressive triple‐negative breast cancer (TNBC).^1^, ^2^ Both early‐onset breast cancer and TNBC are associated with worse breast cancer outcomes^1^ and a higher risk of having hereditary breast cancer (HBC).^1^, ^3^ HBC accounts for approximately 5% to 10% of all breast cancers,^4^, ^5^ most commonly because of germline pathogenic or likely pathogenic variants (GPVs) in *BRCA1* and *BRCA2.* Additional genes, including those of high (e.g., *PALB2*, *TP53*, *PTEN*) and moderate (e.g., *ATM, CHEK2, RAD51C, RAD51D*, *BARD1*) penetrance also contribute.^4^, ^5^

Most studies evaluating HBC genes have focused primarily on women of European ancestry,^4^, ^5^ partly because of low genetic testing rates among populations with other ancestry of geographic origin.^6^ This underuse of genetic testing has been consistently observed across clinical settings, which persists even among women who meet established criteria for testing based on age at diagnosis, tumor subtype, and even family history.^7^, ^8^ Consequently, there remains limited ability to accurately assess frequencies of GPVs by gene and associated clinicopathologic characteristics. Importantly, the disparity in genetic testing rates in combination with the earlier age at diagnosis and higher rates of TNBC among Black women underscore the importance of characterizing HBCs among young Black women.

Current data suggest that women of African ancestry have frequencies of inherited breast cancers comparable to,^9^, ^10^, ^11^ or potentially higher than,^12^, ^13^, ^14^, ^15^, ^16^, ^17^ those observed among women of European ancestry. Prior efforts, predominantly in women of European ancestry, show that HBC GPV frequency increases with younger age at breast cancer diagnosis, and is higher among those with TNBC.^18^ Furthermore, histopathologic patterns vary by gene, with the majority of breast cancers observed in *BRCA1*, *RAD51C*, *RAD51D*, and *BARD1* carriers being TNBC.^18^ Although *BRCA2* and *PALB2* carriers are more likely to have TNBC compared to sporadic cases, the majority of breast cancers are hormone receptor (HR)–positive (estrogen receptor [ER])–positive and/or progesterone receptor [PR]–positive) and HER2‐negative, with increased risks for high‐grade disease.^18^ *ATM* and *CHEK2* carriers with breast cancer predominantly have high‐grade ER‐positive disease.^18^

In this study, we evaluated the demographic and clinicopathologic characteristics of HBC in a cancer registry‐based collection of young Black women with breast cancer, including distribution of GPVs across HBC genes. Among carriers of GPVs, we assessed family history of breast and ovarian cancer, distribution of age at diagnosis, and immunohistochemistry‐based clinical subtypes by gene.

## METHODS

### Study cohort

Eligible participants were Black women diagnosed with invasive breast cancer, ≤age 50 years, between 2005 and 2018, living in Florida or Tennessee at the time of diagnosis. Registry‐based recruitment was initiated on approval by the Institutional Review Boards (IRB) at Vanderbilt University (IRB #170233) and the Departments of Health in Florida (IRB #2011‐05‐VBU) and Tennessee (IRB #2019‐0139). Per previously described state‐mandated procedures,^17^ recruitment methods consisted of initial contact by mail, and, if no response, a phone call to explain the study and determine interest in participation. All participants provided written consent to participate in the study, and all research procedures were conducted in accordance with the Helsinki Declaration. Participants were asked to provide informed consent, sign a medical records and tissue/tumor release, provide a saliva sample for HBC testing, and complete a questionnaire, which collected data on sociodemographic, epidemiologic, and lifestyle factors. Clinical data were collected on all eligible participants who self‐reported Black race, which included those of African American, Afro Caribbean, and other/mixed descent within the recruitment time frame.

### Clinical and demographic variables

Clinical data included age at diagnosis, tumor size, stage, histologic subtype, immunohistochemical (IHC) subtype, and grade. Clinical receptor status (ER, PR, and HER2) was obtained from pathology reports or other medical records, supplemented by cancer registry data and self‐reported questionnaire data. ER and PR positivity was defined as 1% or greater, whereas values <1% were classified as negative. Breast tumors were stratified on receptor status into the following clinical subtypes: HR+ (ER+ and/or PR+)/HER2–; HER2+; and TNBC (ER–, PR–, and HER2–). For participants who reported genetic testing before study enrollment, genetic test reports and medical records were requested to confirm results.

### Germline testing for HBC

The focus of the current analyses was on genes involved in hereditary breast and/or ovarian cancer, including *ATM, BARD1, BRCA1, BRCA2, BRIP1, CDH1, CHEK2, PALB2, RAD51C, RAD51D,* and *TP53.* All of these genes are considered established breast cancer predisposition genes,^3^, ^4^, ^5^ other than *BRIP1*, which is an established ovarian cancer predisposition gene with limited strength of evidence to support an association with breast cancer.^3^, ^19^, ^20^ For participants who provided a saliva sample for germline testing through the study, genes were sequenced using dual bar‐coded QIA‐seq (Qiagen) multiplex amplicon‐based analysis on a custom panel, as previously described.^5^ All study participants were offered genetic testing through the study regardless of whether they had prior clinical testing performed previously, as part of their care. Individuals were considered to have a GPV if they had a loss‐of‐function variant or a variant previously classified as a GPV in the ClinVar database.

### West African ancestry

A saliva sample was collected using an Oragene Self‐Collection kit (DNA Genotek, Inc.) and shipped to the investigators for genomic DNA (gDNA) extraction. NanoDrop and Qubit technologies were used for gDNA quantification and quality assessment. gDNA samples were stored at –80°C before genotyping. Samples were genotyped on either the Infinium OncoArray‐500K (Illumina, Inc.) or Expanded Multi‐Ethnic Genotyping Array (MEGA^EX^) (Illumina, Inc.). Standard sample‐ and variant‐level quality control procedures were performed. Ancestry proportions for each individual were estimated from multilocus single nucleotide polymorphism genotype data using the maximum likelihood‐based ADMIXTURE method,^21^ using the Globe13 calculator from the Dodecad Ancestry project^22^ as reference, as described in previously published work.^23^

### Statistical analysis

Participant characteristics and HBC GPV results were summarized with descriptive statistics, and those with GPVs in HBC genes were compared to sporadic cases based on demographic, clinical, and histopathological variables. Continuous variables were summarized with means and SDs and compared using Wilcoxon rank‐sum tests. Categorical variables were summarized using frequencies and percentages and compared using Pearson chi‐squared tests when number of carriers was 5 or more, and Fisher exact tests were used when expected counts were smaller than 5. Percentages were determined using only known counts for each variable, excluding any unknown values. For gene‐specific comparisons, each HBC gene was independently compared with sporadic cases. Fisher exact tests were used for these comparisons, with Monte Carlo simulation applied when expected counts were small.

Additional analyses stratified by HBC genes compared to sporadic cases included age at diagnosis, tumor size, tumor grade, stage, histology, IHC subtype, West African genetic ancestry, and family history of breast and ovarian cancer. All statistical tests were two‐sided, and *p* values <.05 were considered statistically significant.

## RESULTS

**FIGURE 1:**
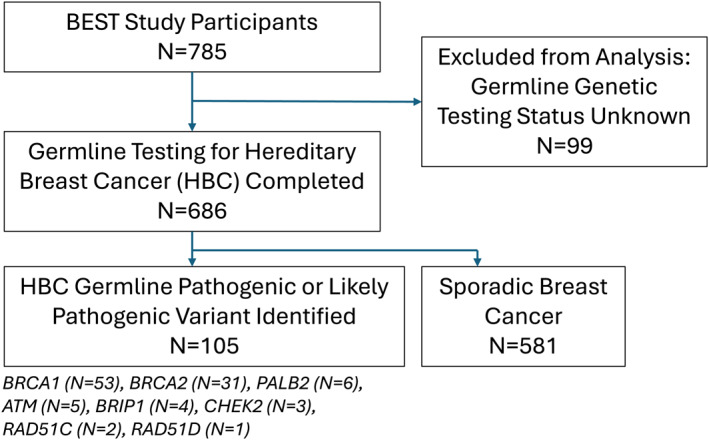
Overview of study population.

A total of 785 women with invasive breast cancer were included in this study (Table 1). The mean age at diagnosis was 42.3 years (21–50 years). More than 25% of women had TNBC, and most presented with nonmetastatic disease (96.4%), invasive ductal carcinoma (81.5%), and high‐grade tumors (57.6%) (Table 1). Of those with confirmed germline testing (*n* = 686), GPVs were detected in 105 (15.3%) participants (Figure 1) in one of the following breast and/or ovarian cancer predisposition genes: *BRCA1* (*n* = 53), *BRCA2* (*n* = 31), *PALB2* (*n* = 6), *ATM* (*n* = 5), *BRIP1* (*n* = 4), *CHEK2* (*n* = 3), *RAD51C* (*n* = 2), and *RAD51D* (*n* = 1). The mean percentage of West African ancestry by gene ranged from 61% in *CHEK2* carriers to 79% in *RAD51D* carriers (Table 2).

**TABLE 1:** Description of the study population.

Characteristic	Total cohort	
	*N* = 785	
	No.	%
Age at diagnosis		
≤40	264	33.6
41–45	224	28.5
46–50	297	37.8
Histology		
Ductal	627	81.5
Lobular	47	6.1
Mixed	15	2.0
Other	80	10.4
Unknown	16	
Tumor grade		
Grade I	74	9.9
Grade II	244	32.5
Grade III	432	57.6
Unknown	35	
Stage		
Localized	294	38.9
Regional	434	57.5
Distant	27	3.6
Unknown	30	
Subtype		
HR+, HER2–	374	52.3
HER2+	146	20.4
TNBC	195	27.3
Unknown	70	
First‐degree family history of breast cancer		
No	553	77.8
Yes	158	22.2
Unknown	74	
Second‐degree family history of breast cancer		
No	423	59.5
Yes	288	40.5
Unknown	74	
First‐degree family history of ovarian cancer		
No	686	96.6
Yes	24	3.4
Unknown	75	
Second‐degree family history of ovarian cancer		
No	633	89.5%
Yes	74	10.5%
Unknown	78	

Abbreviation: TNBC, triple‐negative breast cancer.

**TABLE 2:** Frequency of germline pathogenic/likely pathogenic variants and West African ancestry.

Gene	N	% of cohort	% of carriers	Mean %
		*N* = 686	*N* = 105	West African ancestry
				*N* = 561^a^
Sporadic	581	84.7	N/A	74
Germline	105	15.3	N/A	72
*BRCA1*	53	7.7	50.5%	72
*BRCA2*	31	4.5	29.5%	73
*PALB2*	6	0.9	5.7%	74
*ATM*	5	0.7	4.8%	69
*BRIP1*	4	0.6	3.8%	70
*CHEK2*	3	0.4	2.9%	61
*RAD51C*	2	0.3	1.9%	76
*RAD51D*	1	0.1	1.0%	79

Abbreviation: N/A, not available.

^a^ 11 *BRCA1* carriers, 4 *BRCA2* carriers, 2 *BRIP1* carriers, 1 *CHEK2* carrier, and 107 sporadic cases without ancestry data available.

To determine gene‐specific clinicopathological associations, we compared clinical characteristics of the sporadic group (*n* = 581) with each gene‐specific group for which there were five or more GPV carriers identified (i.e., *BRCA1, BRCA2, PALB2,* and *ATM*), as presented in Table 3. Compared to sporadic cases, women with *BRCA1* GPVs were significantly more likely to be diagnosed at a younger age (*p* < .01) and have high‐grade disease (*p* < .01). Additionally, *BRCA1* carriers were significantly more likely to report a family history of breast cancer among first‐ and second‐degree relatives (*p* < .001), whereas *BRCA2* and *PALB2* carriers were more likely to report family history of breast cancer among second degree relatives (*p* = .02 and .03, respectively) when compared to sporadic cases.

**TABLE 3:** Demographic and clinical characteristics (genes with >5 germline pathogenic/likely pathogenic variants).

	*ATM*		*BRCA1*		*BRCA2*		*PALB2*		Sporadic
	*N* = 5		*N* = 53		*N* = 31		*N* = 6		*N* = 581
	*n* (%)	*p*	*n* (%)	*p*	*n* (%)	*p*	*n* (%)	*p*	*n* (%)
Age at diagnosis (mean)	42	.91	39	<.01	41	.13	42	.83	43
Tumor size (cm) (mean)	2.7	.71	2.3	.79	2.6	.67	4.8	.17	2.4
Histology		1.00		.15		.80		.37	
Ductal	5 (100%)		46 (87%)		24 (77%)		3 (75%)		454 (80%)
Lobular	‐		‐		3 (10%)		1 (25%)		42 (7%)
Mixed	‐		1 (2%)		‐		‐		9 (2%)
Other	‐		6 (11%)		4 (13%)		‐		64 (11%)
Unknown	‐		‐		‐		2		12
Tumor grade		1.00		<.01		.27		1.00	
Grade I	‐		‐		2 (7%)		‐		64 (12%)
Grade II	2 (40%)		7 (14%)		7 (23%)		2 (40%)		192 (35%)
Grade III	3 (60%)		43 (86%)		21 (70%)		3 (60%)		299 (54%)
Unknown	‐		3		1		1		26
Stage		.09		.59		.34		1.00	
Localized	1 (20%)		17 (33%)		11 (37%)		2 (33%)		228 (40%)
Regional	3 (60%)		34 (65%)		17 (57%)		4 (67%)		330 (58%)
Distant	1 (20%)		1 (2%)		2 (7%)		‐		15 (3%)
Unknown	‐		1		1		‐		8
First‐degree family history of breast cancer		1.00		<.001		.36		1.00	
No	4 (80%)		23 (44%)		23 (74%)		5 (83%)		460 (80%)
Yes	1 (20%)		29 (56%)		8 (26%)		1 (17%)		112 (20%)
Unknown	‐		1		‐		‐		9
Second‐degree family history of breast cancer		.66		<.001		.02		.03	
No	4 (80%)		16 (31%)		13 (42%)		1 (17%)		362 (63%)
Yes	1 (20%)		36 (69%)		18 (58%)		5 (83%)		210 (37%)
Unknown	‐		1		‐		‐		9
First‐degree family history of ovarian cancer		1.00		0.30		1.00		.17	
No	5 (100%)		47 (90%)		31 (100%)		5 (83%)		557 (98%)
Yes	‐		5 (10%)		‐		1 (17%)		14 (2%)
Unknown	‐		1		‐		‐		10
Second‐degree family history of ovarian cancer		1.00		0.02		.35		.47	
No	5 (100%)		41 (79%)		29 (97%)		5 (83%)		514 (90%)
Yes	‐		11 (21%)		1 (3%)		1 (17%)		55 (10%)
Unknown	‐		1		1		‐		12

Most breast cancers (52.8%) were diagnosed at or below age 40 years in *BRCA1* carriers, whereas other HBC genes had similar distributions across age groups (Table 4). TNBCs were overrepresented among *BRCA1* carriers (75.5%) compared to sporadic cases (23.6%) (Table 5). Among HR+/HER2– breast cancers and TNBCs, the proportion with HBC GPVs were 35/329 (10.6%) and 50/176 (28.4%), respectively. ER status varied by HBC gene, with the majority of *BRCA1*‐associated tumors being ER‐negative (73.6%), whereas *BRCA2*‐ and *PALB2*‐associated tumors were predominantly ER+ (58.1% and 66.7%, respectively). *ATM‐* and *CHEK2*‐associated tumors were all ER+. The distributions of ER+ and ER– tumors across gene groups are presented in Table 5. Among ER+ and ER– breast cancers, the proportion with HBC GPVs were 48/429 (11.2%) and 57/236 (24.2%), respectively.

**TABLE 4:** Age distribution by gene.

Gene	N	*n* ≤ 40 (%)	*n* = 41–45 (%)	*n* = 46–50 (%)
Sporadic	581	184 (31.7%)	172 (29.6%)	225 (38.7%)
Germline	105	49 (46.6%)	25 (23.8%)	31 (29.5%)
*BRCA1*	53	28 (52.8%)	12 (22.6%)	13 (24.5%)
*BRCA2*	31	12 (38.7%)	11 (35.5%)	8 (25.8%)
*PALB2*	6	3 (50.0%)	‐	3 (50.0%)
*ATM*	5	2 (40.0%)	1 (20.0%)	2 (40.0%)
*BRIP1*	4	2 (50.0%)	‐	2 (50.0%)
*CHEK2*	3	1 (33.3%)	1 (33.3%)	1 (33.3%)
*RAD51C*	2	1 (50.0%)	‐	1 (50.0%)
*RAD51D*	1	‐	‐	1 (100%)

**TABLE 5:** Histopathology‐based breast cancer subtypes by germline status.

Gene	No.	HR+, HER2–, *N* = 329 (52.0%)	HER2+	TNBC	*p*	ER+	ER–, *N* = 236, *n* (%)
			*N* = 128 (20.0%)	*N* = 176 (28.0%)		*N* = 429, *n* (%)	
Sporadic^a^ ^,^ ^b^	581	294 (55.1%)	114 (21.3%)	126 (23.6%)		381 (68.0%)	179 (32.0%)
Germline	105	35 (35.4%)	14 (14.1%)	50 (50.1%)		48 (45.7%)	57 (54.2%)
*BRCA1* ^a^	53	8 (16.3%)	4 (8.2%)	37 (75.5%)	<.001^d^	14 (26.4%)	39 (73.6%)
*BRCA2* ^a^	31	16 (55.2%)	3 (10.3%)	10 (34.5%)	0.23^d^	18 (58.1%)	13 (41.9%)
*PALB2*	6	3 (50.0%)	2 (33.3%)	1 (16.7%)	0.62^e^	4 (66.7%)	2 (33.3%)
*ATM*	5	2 (40.0%)	3 (60.0%)	‐	0.07^e^	5 (100%)	‐
*BRIP1* ^c^	4	3 (75.0%)	‐	1 (25.0%)	‐	3 (75.0%)	1 (25.0%)
*CHEK2*	3	2 (66.7%)	1 (33.3%)	‐	0.619^e^	3 (100%)	‐
*RAD51C*	2	‐	1 (50.0%)	1 (50.0%)	0.079^e^	‐	2 (100%)
*RAD51D* ^c^	1	1 (100%)	‐	‐	‐	1 (100.0%)	‐

Abbreviation: TNBC, triple‐negative breast cancer.

^a^ 4 *BRCA1* carriers, 2 *BRCA2* carriers, and 47 sporadic cases with unknown subtype.

^b^ 21 sporadic cases with unknown estrogen receptor status.

^c^ *BRIP1* and *RAD51D* had insufficient sample size for hypothesis testing.

^d^ Fisher exact test with Monte Carlo simulation comparing four‐category subtype distributions (HR+/HER2−, HR+/HER2+, HR−/HER2+, TNBC) between the indicated germline gene and sporadic cases.

^e^ Fisher exact test with Monte Carlo simulation comparing binary subtype distributions between the indicated germline gene and sporadic cases, applied when sample size was limited.

## DISCUSSION

This study is among the largest to characterize HBC in comparison to sporadic breast cancers within a registry‐based sample of young Black women diagnosed with breast cancer at age 50 years and younger. The overall frequency of HBC GPVs was 15.3%, with the majority occurring in *BRCA1* and *BRCA2*. Among women with *BRCA1*‐related tumors, there was overrepresentation of high‐grade tumors and TNBC. A family history of breast cancer was more common among *BRCA1*, *BRCA2*, and *PALB2* carriers. Finally, among those with *BRCA1* GPVs, the majority of breast cancers occurred at or below age 40 years, whereas the age at diagnosis was more evenly distributed up to age 50 years for other gene carriers.

The most common genes with GPVs reported in US‐based Black women with breast cancer in prior studies were *BRCA1* and *BRCA2*, followed by *PALB2*,^9^, ^10^ consistent with our findings. However, there have been mixed results reported among studies focused on the proportions of HBC GPVs among Black women with breast cancer. Emerging data suggest rates of *BRCA1* and *BRCA2* GPVs among Black women with HBC in the United States,^12^, ^13^ the Caribbean,^14^, ^15^ and western sub‐Saharan Africa^16^, ^17^ may be higher than that previously reported among predominantly White populations. However, a study of 4000 Black and 25,000 White women across all age groups showed similar overall frequency of GPVs (∼5%) in both populations.^11^ In that study, there was a significantly higher frequency of GPVs observed in high penetrance genes (*BRCA2* and *PALB2*) among Black women, whereas a significantly lower frequency of *CHEK2* GPVs was observed, likely because of the strong founder effect among individuals of European ancestry. Similarly, we also observed a low frequency of *CHEK2* GPVs in our study, with these women having the lowest proportion of West African ancestry compared to the other HBC genes. Consequently, data from our study and others suggest that women of African ancestry with breast cancer have at least similar frequencies of HBC GPVs as individuals of European ancestry, with lower frequencies of *CHEK2* GPVs observed.

Prior studies, including those conducted in Black women, have reported younger ages of breast cancer diagnosis in women with GPVs in *BRCA1* compared to *BRCA2*.^9^, ^10^, ^24^ These findings are consistent with our results, where the mean age of breast cancer was lowest in those with GPVs in *BRCA1*, followed by *BRCA2*, both of which were lower than *PALB2* and *CHEK2.* Regarding family history of breast cancer, prior studies have reported an association with HBC GPVs.^9^, ^25^ In fact, a study of 67,692 women with breast cancer, including 13% Black women, reported a significantly higher proportion with *BRCA1*, *BRCA2*, and *PALB2* GPVs had a family history of breast cancer compared to those with sporadic tumors.^25^ Similarly, among 5054 Black women with breast cancer, family history of breast cancer among women with *BRCA1*, *BRCA2*, or *PALB2* GPVs was higher compared to those with sporadic tumors, with odds ratios of 9.0, 8.0, and 13.6, respectively.^9^ These findings are consistent with our study, where women with *BRCA1*, *BRCA2*, or *PALB2* GPVs were significantly more likely to have a first‐ and/or second‐degree relative with breast cancer than sporadic cases.

Prior studies involving predominantly women of European ancestry have reported that the frequency of *BRCA1* and *BRCA2* GPVs decreased with age at breast cancer diagnosis, indicating that hereditary influences are strongest in younger women.^18^, ^24^ In the two largest studies of Black women with breast cancer that evaluated HBC GPVs,^9^, ^10^ *BRCA1*‐associated breast cancers occurred at younger ages than *BRCA2*‐associated cancers, whereas *PALB2, ATM,* and *CHEK2* showed age distributions spanning both younger and older groups, similar to our findings.

GPV frequencies reported in HR+/HER2– breast cancers and TNBC have been reported as 8.6% and 17.2%, respectively.^24^ In our study, we observed similar rates in HR+/HER2– breast cancers (10.6%), but higher rates in TNBC (28.4%), likely because of our exclusive focus on young women with breast cancer. The frequency of HBC GPVs reported in prior studies among predominantly White women with breast cancer across all ages has been higher among ER– cases (8.2%) compared to ER+ cases (3.0%).^5^ Similarly, among Black women with breast cancer across all ages, HBC GPV frequencies have been higher among ER– (8.9%‐11.5%) compared to ER+ (5.0%‐6.4%) cases.^9^, ^10^ However, these prior studies did not present HBC GPV prevalence estimates by age, limiting direct comparisons. Therefore, although we also found higher HBC GPV frequencies among ER– cases (24.2%), compared to ER+ cases (11.2%), our observed frequencies are likely higher because of our focus exclusively on young women, in which HBC GPVs are enriched compared to older age groups.

HBCs exhibit distinct histopathologic patterns that vary by gene, most of which have been studied in European ancestry populations.^18^, ^20^ Our findings related to IHC‐based subtypes across genes were overall consistent with those reported in prior studies of predominantly women of European ancestry,^4^, ^5^, ^18^ in which TNBC accounted for the majority of *BRCA1*‐associated breast cancers. The largest study to date to evaluate breast cancer characteristics in HBC was based on 42,680 women with breast cancer.^18^ Among *BRCA1*‐associated breast cancers in this study, TNBC has accounted for 69.2% in women younger than age 40 years and 59.5% across all ages, which is consistent with our study where TNBC was observed in 75.5%. Furthermore, we observed similar rates of *BRCA2‐*associated breast cancer in HR+/HER2– (55.2%) and TNBC (34.5%) as prior studies that reported enrichment in HR+/HER2– (65%‐70%), and TNBC (30%‐40%) compared to sporadic cancers.^4^, ^5^ Similarly, *PALB2*‐associated breast cancers are predominantly ER+/HER2–,^18^, ^20^ consistent with our findings, albeit based on a very small sample size, limiting our ability to draw any conclusions. Finally, the majority of *ATM*‐associated breast cancers are HR+,^18^, ^24^ which is consistent with our study; however, most of these tumors are generally not HER2+, in contrast to our study where 60% of tumors were HER2+. However, these findings are based on a small sample size, and it is possible that the young age of our cohort contributed to this observation.

Collectively, our data indicate that demographic and clinicopathologic breast cancer characteristics observed in young Black women are comparable to those seen among White women, highlighting the importance of genetic testing across all populations. Importantly, the underuse of genetic testing among Black women is due to patient, provider, and system‐level factors,^7^ and remains an unmet clinical need. Consequently, our findings underscore the need to improve testing rates so that all populations may benefit from this information.

Our study has several strengths, including the large sample size of unselected Black women diagnosed with breast cancer at or below age 50 years, making it unique compared to other cohorts, which generally are not focused exclusively on young patients with breast cancer. Consequently, our observed frequencies of GPVs need to be interpreted in the context of our young cohort, and results may not be generalizable to later‐onset breast cancers. Furthermore, free genetic testing was offered to all study participants, including those who reported prior testing, removing the barrier to access testing and demonstrating that no‐cost testing may lead to higher completion of testing. Despite these strengths, there remain some limitations given that participation required consent, which could introduce a selection bias. Moreover, despite our large sample of young Black women, the absolute numbers of those with GPVs for many susceptibility genes remain small, limiting the precision of gene‐level estimates and associated characteristics.

Overall, our study is among the largest single studies of unselected young Black women with invasive breast cancer to characterize HBCs relative to sporadic tumors. Our study identified a high prevalence of HBC GPVs (15.3%), with overrepresentation of TNBCs and HR+/HER2– breast cancers among those with *BRCA1* and *ATM* GPVs, respectively. Family history of breast cancer was more common among women with *BRCA1*, *BRCA2*, and *PALB2* GPVs, whereas a higher frequency of *BRCA1* and *BRCA2* GPVs was seen at earlier ages of diagnosis. Our findings contribute to characterization of HBCs in Black women, highlighting the importance of genetic testing in this underserved population. These women have a higher prevalence of early‐onset breast cancer yet lower genetic testing rates. Ultimately, there remains a need to address disparities in this undertested population, so those with an HBC GPV can benefit from the latest treatment advances, reduce their risks of future cancer, and share this information with at‐risk family members.

## AUTHOR CONTRIBUTIONS

**Heather K. Beasley**: Writing—original draft; formal analysis; writing—review and editing; conceptualization. **Tiana Shah**: Writing—original draft; writing—review and editing; conceptualization. **Rory J. Tinker**: Formal analysis; methodology; writing—review and editing; conceptualization. **Anne Weidner**: Writing—review and editing; project administration; data curation; formal analysis. **Lindsay Venton**: Data curation; writing—review and editing; project administration. **Chunling Hu**: Writing—review and editing; data curation. **Mya L. Roberson**: Writing—review and editing. **Brian D. Lehmann**: Writing—review and editing; formal analysis; supervision. **Fergus J. Couch**: Writing—review and editing; data curation; funding acquisition; resources. **Sonya Reid**: Writing—review and editing; funding acquisition; supervision; conceptualization; investigation; resources; data curation; methodology. **Kelly Metcalfe**: Conceptualization; writing—review and editing. **Tuya Pal**: Conceptualization; investigation; funding acquisition; writing—original draft; project administration; supervision; data curation; writing—review and editing; formal analysis; resources; methodology.

## CONFLICT OF INTEREST STATEMENT

None.

## ETHICS APPROVAL STATEMENT

Black Women with Breast Cancer: Etiology, Survival, and Treatment Outcomes (BEST) Study (Vanderbilt IRB# 170233)

## FUNDING STATEMENT

This work has been supported in part by NCI grant R01CA204819 (T.P.), Susan G. Komen SAC210105 (T.P.), Breast Cancer Research Foundation grant HEI‐25‐011 (T.P., S.R.), Nashville Wine Auction (T.P., S.R.), American Association for Cancer Research in Partnership with Victoria’s Secret and Pelotonia Career Development Award 22‐20‐73‐ROBE (M.L.R.), NIH/NCI grants R35CA253187, U24CA258058, P50CA116201, and the Breast Cancer Research Foundation BCRF‐030‐24 (F.C.), DOD/BCRP, BC201286 (B.D.L.), Burroughs Wellcome Fund PDEP, G‐1022376, and NIH T32 Training Fellowship 5T32GM7569‐49 (H.K.B.).

## PATIENT CONSENT STATEMENT

Eligible individuals were recruited through the state cancer registries in Tennessee and Florida as part of the Black Women with Breast Cancer: Etiology, Survival, and Treatment Outcomes (BEST) Study. Existing participants in the BEST Study who meet eligibility criteria, with verified test results and a signed tissue/tumor release form, will be included in the proposed study.

## Data Availability

The data that support the findings of this study are available on request from the corresponding author. The data are not publicly available because of privacy or ethical restrictions.
